# Autophagy supports *Candida glabrata* survival during phagocytosis

**DOI:** 10.1111/j.1462-5822.2009.01391.x

**Published:** 2009-10-26

**Authors:** Andreas Roetzer, Nina Gratz, Pavel Kovarik, Christoph Schüller

**Affiliations:** 1 Departments of Biochemistry and Cell Biology, Max F. Perutz Laboratories, University of ViennaA-1030 Vienna, Austria; 2 Departments of Microbiology and Immunology, Max F. Perutz Laboratories, University of ViennaA-1030 Vienna, Austria

## Abstract

The opportunistic human fungal pathogen *Candida glabrata* is confronted with phagocytic cells of the host defence system. Survival of internalized cells is thought to contribute to successful dissemination. We investigated the reaction of engulfed *C. glabrata* cells using fluorescent protein fusions of the transcription factors CgYap1 and CgMig1 and catalase CgCta1. The expression level and peroxisomal localization of catalase was used to monitor the metabolic and stress status of internalized *C. glabrata* cells. These reporters revealed that the phagocytosed *C. glabrata* cells were exposed to transient oxidative stress and starved for carbon source. Cells trapped within macrophages increased their peroxisome numbers indicating a metabolic switch. Prolonged phagocytosis caused a pexophagy-mediated decline in peroxisome numbers. Autophagy, and in particular pexophagy, contributed to survival of *C. glabrata* during engulfment. Mutants lacking *CgATG11* or *CgATG17*, genes required for pexophagy and non-selective autophagy, respectively, displayed reduced survival rates. Furthermore, both CgAtg11 and CgAtg17 contribute to survival, since the double mutant was highly sensitive to engulfment. Inhibition of peroxisome formation by deletion of *CgPEX3* partially restored viability of Cg*ATG11* deletion mutants during engulfment. This suggests that peroxisome formation and maintenance might sequester resources required for optimal survival. Mobilization of intracellular resources via autophagy is an important virulence factor that supports the viability of *C. glabrata* in the phagosomal compartment of infected innate immune cells.

## Introduction

*Candida glabrata* belongs to the diverse group of human fungal pathogens and is phylogenetically closely related to *Saccharomyces cerevisiae* (Kaur *et al.*, 2005, Marcet-Houben and Gabaldon, 2009). The high similarity of *C. glabrata* to *S. cerevisiae* suggests that also for fungi, relatively small genetic changes may be sufficient for adaptation to a pathogenic lifestyle (Dujon *et al.*, 2004). *C. glabrata* is a common commensal, but can turn into an opportunistic pathogen with a rising frequency of isolates among immunocompromised patients and elder people (Li *et al.*, 2007; Pfaller and Diekema, 2007; Presterl *et al.*, 2007). In the host environment, *C. glabrata* has to evade or survive attacks of the cell-mediated immune system (Nicola *et al.*, 2008). Counterstrategies of fungal pathogens differ between species. *Candida albicans* destroys macrophages by hyphal outgrowth. Alternatively, *Cryptococcus neoformans* either lyses macrophages or escapes via phagosomal extrusion (Alvarez and Casadevall, 2006; Ma *et al.*, 2006). *C. glabrata* engulfed by macrophages do not undergo morphological transitions such as *C. albicans* (Leberer *et al.*, 2001). An open question concerns how *C. glabrata* is coping with cells of the immune system, such as macrophages.

The phagosome is a hostile environment for fungi (reviewed in Nicola *et al.*, 2008). After internalization of microbial cells, the organelle maturates into the phagolysosome containing mature hydrolytic enzymes and a more acidic pH 4.5–5.5 (Geisow *et al.*, 1981; Levitz *et al.*, 1999). Additionally, the NADPH oxidase complex generates reactive oxidative species to attack internalized microorganisms (for review see Romani, 2004; Segal, 2005). Thus, commensal and pathogenic fungi are exposed to reactive oxygen species (ROS) produced by polymorphonuclear leucocytes, macrophages and dendritic cells (Miller and Britigan, 1997; Missall *et al.*, 2004; Gildea *et al.*, 2005). On the fungal side, antioxidant defence enzymes such as catalase, superoxide dismutase, thioredoxin- and glutathione-dependent peroxidases and reductases guard against oxidative stress and are thus considered virulence factors (Johnson *et al.*, 2002; Cox *et al.*, 2003; Missall *et al.*, 2004; Chaves *et al.*, 2007). Oxidative stress as defence strategy is not restricted to combat fungal infections. Invading bacteria, such as *Staphylococcus aureus*, or the malaria parasites *Plasmodium* sp., face ROS stress upon engulfment (Becker *et al.*, 2004; Voyich *et al.*, 2005). ROS sensed by microbes act also as signalling molecules. The *C. albicans* catalase, an enzyme which decomposes hydrogen peroxide, has been investigated more closely. *C. albicans* induces catalase when engulfed in neutrophils or in macrophages (Rubin-Bejerano *et al.*, 2003; Lorenz *et al.*, 2004; Enjalbert *et al.*, 2007). Moreover, hydrogen peroxide promotes the morphological transition of *C. albicans* cells to hyphal growth, a form invading the host tissue (Nakagawa, 2008; Nasution *et al.*, 2008). Finally, *C. albicans* devoid of catalase was eliminated more efficiently in a mouse infection model (Nakagawa *et al.*, 2003). The filamentous fungus *Aspergillus fumigatus* lacking the catalases expressed in the mycelium exhibited delayed infection in a rat model of invasive aspergillosis (Paris *et al.*, 2003). In contrast, *C. glabrata* catalase was not a virulence determinant in an immunocompromised mouse model (Cuellar-Cruz *et al.*, 2008). In mice infected with a *C. neoformans* mutant devoid of all four catalases, mortality was unchanged (Giles *et al.*, 2006). Thus the relative importance of individual ROS scavenging enzymes varies between fungal pathogens.

Besides being exposed to oxidative stress, cells engulfed by macrophages adjust their metabolic programme (Fan *et al.*, 2005; Barelle *et al.*, 2006). Engulfed *C. albicans* cells induce many genes involved in non-fermentative carbon metabolism (Prigneau *et al.*, 2003; Lorenz *et al.*, 2004). Phagocytosed *C. glabrata* induces genes encoding enzymes involved in β-oxidation, the glyoxylate cycle and gluconeogenesis (Kaur *et al.*, 2007). Moreover, the glyoxylate cycle, which is required to channel fatty acid-derived two carbon units into metabolism, was early recognized as a virulence determinant for *C. albicans* (Lorenz and Fink, 2001). Other human fungal pathogens also induce glyoxylate cycle components during infection conditions (Rude *et al.*, 2002; Canovas and Andrianopoulos, 2006; Derengowski *et al.*, 2008). However, *A. fumigatus* and *C. neoformans* do not require the glyoxylate cycle for virulence (Idnurm *et al.*, 2007; Schöbel *et al.*, 2007). Some of the enzymes of the glyoxylate cycle are localized in the peroxisomal matrix (for review see, e.g. Kunze *et al.*, 2006). Peroxisomes are inducible, single-membrane organelles which harbour enzymes for the oxidative catabolism of fatty acids, the glyoxylate cycle and others. Generally, peroxisome number and size vary according to metabolic needs (for review see Yan *et al.*, 2005; Platta and Erdmann, 2007).

Autophagy continuously recycles almost all constituents of the cell (for review see Mizushima and Klionsky, 2007; Kraft *et al.*, 2009). Different types of autophagy help organisms to overcome periods of nutrient starvation by recycling intracellular components to sustain vital cellular functions. It seems to be linked to the unique niches and morphology of fungal pathogens (for review see Palmer *et al.*, 2008). For certain pathogenic fungi, autophagy has been identified as a virulence factor. *C. neoformans* requires an intact autophagy pathway during infection (Hu *et al.*, 2008). Peroxisomes and their contents are delivered to the vacuole by the pexophagy pathway, a specialized form of autophagy (Guan *et al.*, 2001; Kim *et al.*, 2001; Farre and Subramani, 2004). In *S. cerevisiae* selective pexophagy is dependent on Atg11 and partly on Atg17 which is also important for non-selective autophagy (Cheong *et al.*, 2005; Yorimitsu and Klionsky, 2005).

Here we investigated responses of *C. glabrata* during its encounter with the macrophage phagosome compartment from which it cannot escape. We developed *in vivo* reporters to track fungal responses to this environment. To detect oxidative and glucose starvation stress of cells, we used fluorescent protein fusions of the *C. glabrata* orthologues of the *S. cerevisiae* transcription factors Yap1 and Mig1 (Kuge *et al.*, 1997; Carlson, 1999). We found the *C. glabrata* catalase gene *CgCTA1* and catalase activity regulated by oxidative stress and glucose starvation. Additionally, we demonstrated GFP–CgCta1 localization to peroxisomes. *C. glabrata* peroxisomes have not been described so far and were here defined by several independent criteria. We found that *C. glabrata* cells engulfed by mouse macrophages experience a mild oxidative stress and sustained carbon starvation. Additionally, peroxisomes became transiently induced in engulfed cells. We explored the role of peroxisomes with various mutants lacking peroxisome biogenesis or autophagy pathways mediating destruction of peroxisomes. We report here that autophagy and, surprisingly, pexophagy is a likely virulence factor for *C. glabrata.* Mutants lacking CgAtg11 and/or CgAtg17 were killed more efficiently by macrophages during engulfment. Thus, for engulfed *C. glabrata* cells, nutrient deprivation represents a serious challenge and mobilization of intracellular resources via autophagy is a major contributor to sustain viability.

## Results

### *C. glabrata* catalase CgCta1 is induced by hydrogen peroxide and carbon starvation

**Fig. 1:**
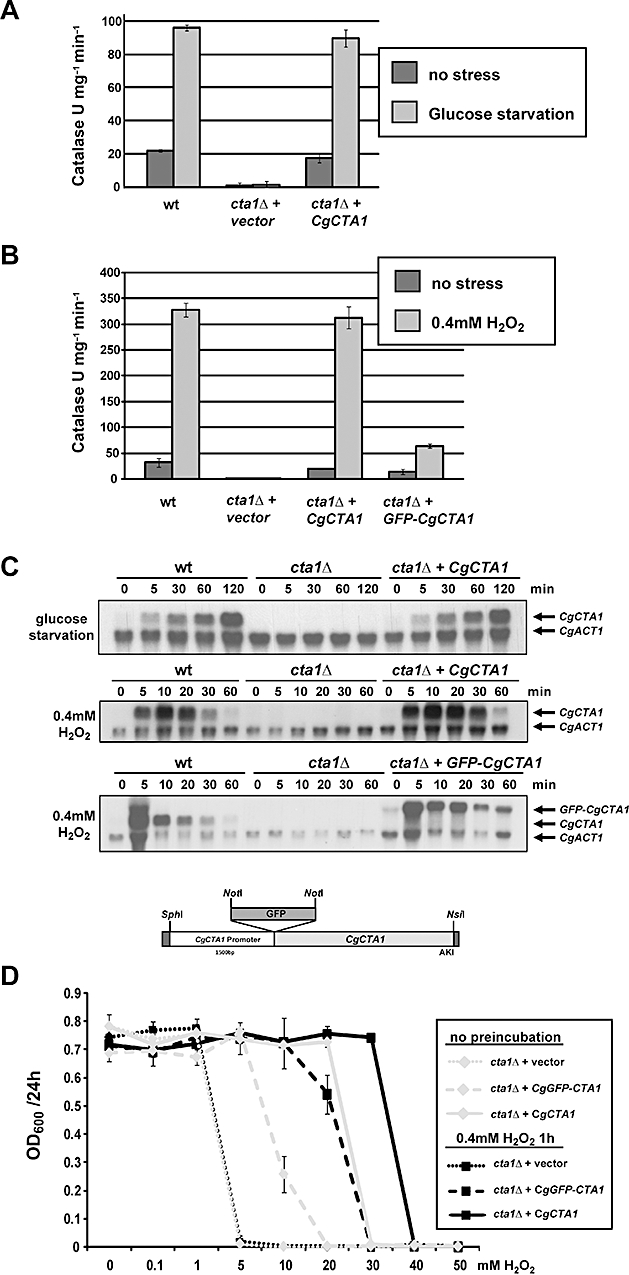
Oxidative stress and carbon source stress regulate *C. glabrata* catalase *CgCTA1*. A. To measure catalase activity upon glucose depletion, cells were grown to log phase in YPD and shifted to medium with 2% or 0.1% glucose and grown for 4 h. Catalase activity was determined as described in *Experimental procedures*. B. Cells were incubated in YPD with 0.4 mM H_2_O_2_ for 45 min. Crude cell extracts were prepared and then assayed for catalase activity. C. Northern blot analysis of *CgCTA1* mRNA levels from wild type, ARCg *cta1*Δ mutant and complemented mutant strain was performed under stress conditions (glucose starvation, 0.4 mM H_2_O_2_). Samples were taken at the indicated time points. *CgACT1* mRNA levels were used as loading control. mRNA levels were visualized by hybridization of radioactive probes and autoradiography. The p*CgC–GFP–CgCTA1* construct with GFP inserted at the N-terminus is illustrated. D. *C. glabrata* ARCg *cta1*Δ mutant complemented with p*GFP–CgCTA1*, p*CgCTA1* or an empty plasmid was grown in synthetic medium to log phase, adjusted to 10^5^ cells ml^−1^ and exposed to indicated doses of hydrogen peroxide. Optical density after 24 h of incubation at 37°C is indicated.

*Candida glabrata* harbours one catalase gene (*CgCTA1*, CAGL0K10868g), related to the yeast peroxisomal catalase *CTA1* gene. The two catalase genes of *S. cerevisiae* are regulated differently. *CTT1*, coding for the cytoplasmic catalase, is induced by stress conditions (Marchler *et al.*, 1993). The *CTA1* gene is expressed only during growth on non-fermentable carbon sources (Cohen *et al.*, 1985; Hartig and Ruis, 1986). To find out the regulatory pattern of the *C. glabrata* catalase, we assayed its activity in crude protein extracts from cells grown either on glucose or on a non-fermentable carbon source. Cells adapting to ethanol as carbon source showed a substantial induction of catalase activity (Fig. 1A). Moreover, mild oxidative stress of 0.4 mM H_2_O_2_ induced *C. glabrata* catalase activity about 10-fold suggesting regulation by both glucose starvation and oxidative stress (Fig. 1B). To verify if the *CgCTA1* gene encodes the only catalase activity in *C. glabrata*, we replaced the open reading frame (ORF) with the *S. cerevisiae URA3* gene (Fig. S1). Catalase activity was undetectable in extracts derived from the mutant strain (Fig. 1A and B). A centromeric plasmid (p*CgCTA1*) harbouring the *CgCTA1* ORF including a 1.8 kb upstream region fully restored wild-type level catalase activity to the *cta1*Δ mutant (Fig. 1A and B). To demonstrate that the regulation of catalase activity occurs at the level of transcription, *CgCTA1* mRNA levels were analysed from cells shifted to medium lacking glucose or exposed to 0.4 mM H_2_O_2_. Glucose-starved cells displayed a continuous increase of *CgCTA1* mRNA immediately after shift to glucose-free medium (Fig. 1C). Hydrogen peroxide stress caused a rapid increase within 10 min. Taken together, regulation of the *C. glabrata* catalase gene *CgCTA1* by carbon source availability and oxidative stress combines elements of both *S. cerevisiae* catalases.

### CgCta1 confers hydrogen peroxide stress resistance

The similarity of the *CgCTA1* gene to the *S. cerevisiae CTA1* gene suggested its peroxisomal localization. To clarify the intracellular localization, we fused a green fluorescent protein (GFP) to the CgCta1 N-terminus (*GFP–CgCTA1*) to preserve the putative peroxisomal targeting sequence 1 (PTS1) (Fig. 1C, lower panel). The preceding 1.8 kb of the *CgCTA1* 5′ untranslated region conferred a wild type-like expression pattern in hydrogen peroxide-stressed cells (Fig. 1C). Basal catalase activity of GFP–CgCta1 was detectable in unstressed cells, whereas hydrogen peroxide stress-induced activity was reduced to about 20% of the wild-type level (Fig. 1B).

We assessed if the reduced activity of GFP–Cta1 interferes with hydrogen peroxide stress resistance. *C. glabrata cta1*Δ mutant cells transformed with either p*GFP–CgCTA1*, p*CgCTA1* or the empty plasmid (p*ACT*) were grown in synthetic medium, the cultures were split and one part treated with 0.4 mM H_2_O_2_. Both were subsequently exposed to higher doses of hydrogen peroxide. Growth was scored after 24 h (Fig. 1D). The *cta1*Δ mutant cells containing the empty plasmid failed to grow in medium containing 5 mM H_2_O_2_. In contrast, the strain carrying the p*CgCTA1* plasmid was resistant to medium supplemented with up to 20 mM H_2_O_2_, whereas pre-incubation with 0.4 mM H_2_O_2_ pushed the growth limit to 40 mM H_2_O_2_, similar to the wild-type parent strain (ΔHTU). Cells expressing the GFP–CgCta1 derivative displayed lower basal resistance. However, naïve cells without pre-treatment tolerated 5 mM H_2_O_2_ and failed to grow only at about 20 mM H_2_O_2_. Pre-treatment with 0.4 mM H_2_O_2_ shifted tolerance to about 30 mM H_2_O_2_. Thus, the GFP-tagged CgCta1 derivative, when compared with the untagged version, conferred resistance to oxidative stress to reduced but overall high level. These results suggested that H_2_O_2_ stress resistance of strains carrying the plasmid-encoded catalase derivatives encompasses the oxidative stress load of 0.4 mM H_2_O_2_ determined for the *in vivo* situation (Enjalbert *et al.*, 2007). Our data also showed that the *C. glabrata* strains tolerated a substantial higher oxidative stress load compared with *S. cerevisiae* laboratory strains, which failed to grow at concentrations higher than 3 mM H_2_O_2_ (Davies *et al.*, 1995; Cuellar-Cruz *et al.*, 2008).

### Localization of GFP–CgCta1 is dependent on the carbon source

**Fig. 2:**
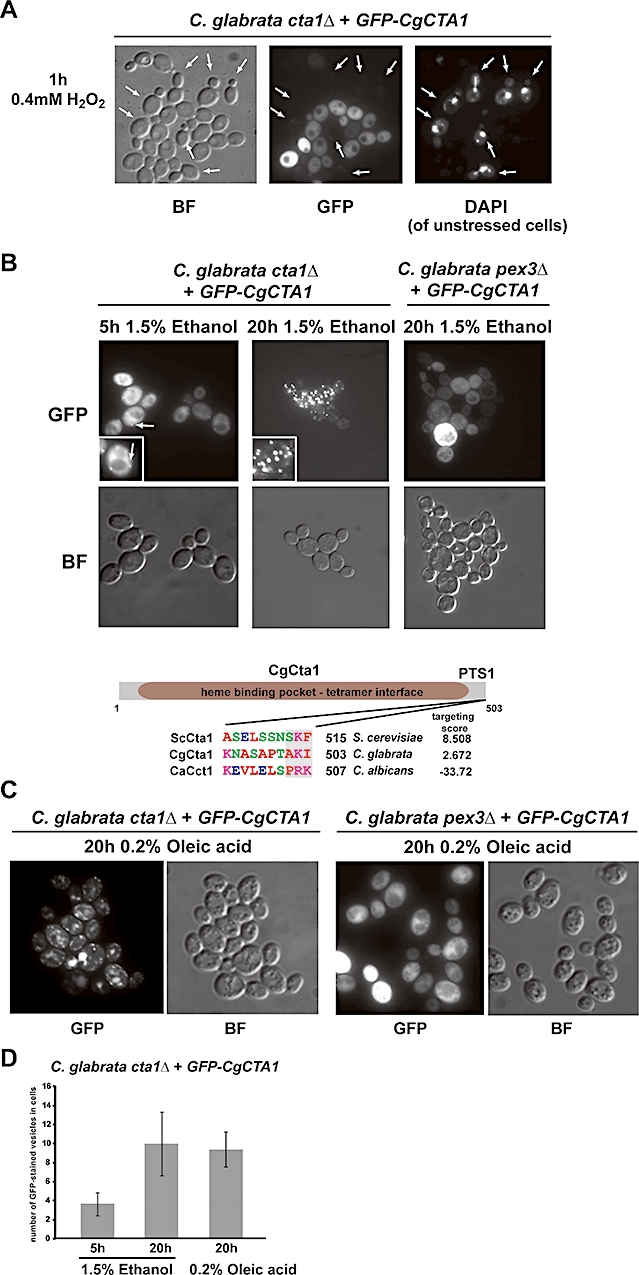
Intracellular localization of *C. glabrata* catalase. A. Localization of GFP–CgCta1 was determined by fluorescence microscopy in ARCg *cta1*Δ cells transformed with p*CgCTA1–GFP–CgCTA1*. Cells were incubated for 1 h after induction of oxidative stress with 0.4 mM H_2_O_2_. Unstressed cells were stained with DAPI (2 μg ml^−1^) for 10 min. Aliquots of both cultures were pooled prior to microscopy. White arrows indicate unstressed cells. B. ARCg *cta1*Δ and ARCg *pex3*Δ mutant strains transformed with p*CgC–GFP–CgCTA1* were grown in synthetic medium with 0.5% glucose and 1.5% ethanol for 20 h. White arrows indicate vesicular structures. Inserts show enlarged pictures of single cells. Possible peroxisomal targeting signals 1 (PTS1) detected at the C-terminus of CgCta1, ScCta1 and CaCct1 (Q6FM56, P15202, Q5AAT2; Neuberger *et al.*, 2003). C. Fluorescence signals of strains as in (B) after growth in medium with 0.2% oleic acid for 20 h. D. Number of peroxisomes in *C. glabrata* cells during growth with ethanol (1.5%) and oleic acid (0.2%) as main carbon source.

Cells expressing *GFP–CgCTA1* were exposed to different stress conditions. In rich medium, GFP–CgCta1 fluorescence was hardly detectable, reflecting its low basal expression of *CgCTA1*. Oxidative stress caused induction of the GFP–CgCta1 fluorescence signal. To compare different expression levels directly, unstressed cells were marked by staining their nucleic acids with DAPI. For microscopy these marked unstressed cells were mixed to cells from the same culture treated for 1 h with 0.4 mM H_2_O_2_. The micrograph demonstrates induction of the fusion protein by oxidative stress and its initial localization in the cytoplasm (Fig. 2A). GFP*–CgCTA1* became also induced after the glucose concentration in the growth medium dropped below 0.03% (Fig. S2A). We then investigated GFP–CgCta1 distribution in cells growing on non-fermentable carbon sources. Cells expressing *GFP–CgCTA1* were grown in medium supplemented with 0.5% glucose and 1.5% ethanol. After 5 h, glucose was exhausted, and cells were switching to the non-fermentable carbon source. (Fig. 2B, left panel). Although the vast majority of GFP–CgCta1 was still located in the cytoplasm, small vesicles accumulating catalase became visible (see insert). After 20 h, almost all GFP–CgCta1 was accumulated in vesicular structures (Fig. 2B, middle panel).

### CgCta1 can localize to peroxisomes

We suspected that the vesicles accumulating GFP–CgCta1 were peroxisomes. The PTS1 of CgCta1 was a boundary case compared with *S. cerevisiae* Cta1 (Fig. 2B, lower panel). To interfere with *C. glabrata* peroxisome assembly, we chose to eliminate the *CgPEX3* gene (CAGL0M01342g). The *S. cerevisiae* orthologue Pex3 has an essential function for peroxisome biogenesis (Hohfeld *et al.*, 1991). The *CgPEX3* ORF was replaced with the *ScURA3* gene and the correct integration was tested by Southern blot (Fig. S1). In these *pex3*Δ mutant cells, GFP–CgCta1 remained distributed in the cytoplasm, even in 1.5% ethanol grown cells (Fig. 2B, right panel). With oleic acid as sole carbon source, *S. cerevisiae* cells increase number and size of peroxisomes (Thieringer *et al.*, 1991). GFP–CgCta1 accumulated in vesicles in cells growing in medium containing 0.2% oleic acid, whereas in *pex3*Δ mutant cells fluorescence was dispersed in the cytoplasm (Fig. 2C)*.* The number of stained vesicles also increased substantially in cells growing on a non-fermentative carbon source (1.5% ethanol) (Fig. 2D). These data suggest that *C. glabrata* accumulates GFP–CgCta1 in CgPex3-dependent structures resembling peroxisomes.

**Fig. 3:**
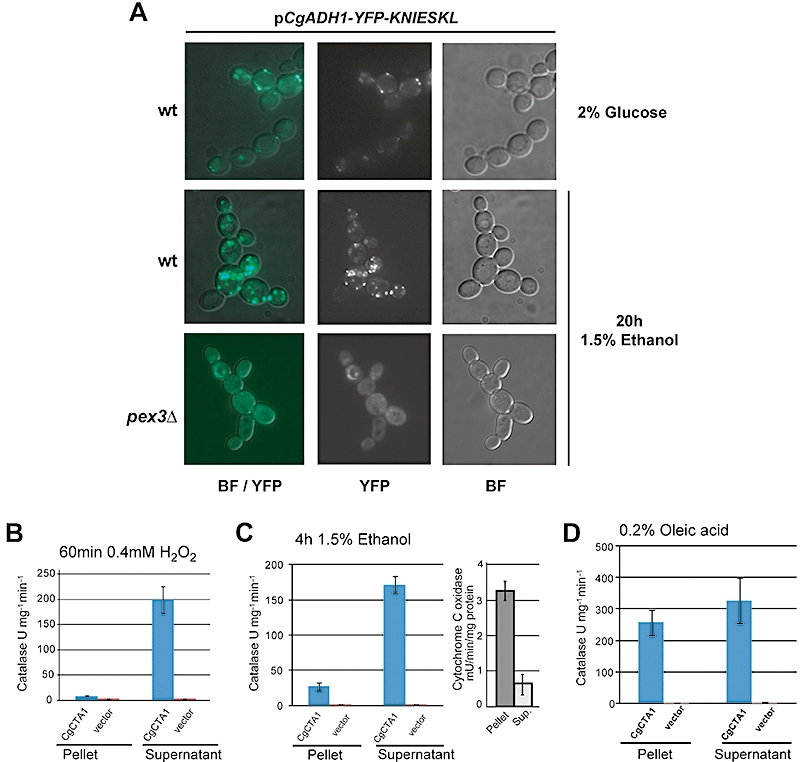
CgCta1 localizes to peroxisomes upon glucose depletion. A. *C. glabrata*ΔHTU and ARCg *pex3*Δ mutant cells expressing YFP–KNIESKL driven by the *CgADH1* promoter were grown in synthetic medium with 2% glucose or 1.5% ethanol for 20 h. Localization of YFP was recorded by fluorescence microscopy and bright field (BF) microscopy. An overlay of YFP and BF microscopy is shown in the left panel. B. The ARCg *cta1*Δ strain carrying pCgC–*CgCTA1* was exposed for 1 h to oxidative stress (0.4 mM H_2_O_2_). Pellets containing mitochondria and small organelles and post-mitochondrial supernatants were assayed for catalase activity. C. The same strain was grown in synthetic medium with 1.5% ethanol for 20 h. Catalase activity was measured in pellets and supernatants. Activity of cytochrome *c* oxidase was measured in pellet and supernatant fractions as described in *Experimental procedures* (right panel). D. Catalase activity in pellets and supernatant fraction collected from ARCg *cta1*Δ containing p*CgC*–*CgCTA1* grown in synthetic medium with 0.2% oleic acid for 20 h.

To visualize peroxisomal structures in *C. glabrata*, we fused a generic peroxisomal targeting signal peptide (KNIESKL) derived from the *S. cerevisiae* citrate synthase to the C-terminus of YFP (Lewin *et al.*, 1990; Kragler *et al.*, 1993). The *YFP–KNIESKL* fusion gene expression was driven by the strong *CgADH1* promoter. YFP fluorescence marked peroxisomes, which increased their number during growth on ethanol (Fig. 3A, upper panel) and were absent in *pex3*Δ mutant cells (Fig. 3A, lower panel). This result confirmed the requirement of CgPex3 for *C. glabrata* peroxisome biogenesis.

The above results indicated a partial organellar localization of catalase, depending on the type of carbon source. To show this, we prepared cell extracts of *cta1*Δ mutant cells expressing *CgCTA1.* We separated these in an organellar pellet and a cytosolic supernatant fraction by centrifugation and tested the fractions for catalase activity. After oxidative stress, the entire induced catalase activity was found in the cytoplasmic supernatant (Fig. 3B). In extracts from cells growing with ethanol as main carbon source, catalase activity was found in the cytosolic supernatant, but about one-fourth of total activity was present in the pellet fraction (Fig. 3C, left panel). To confirm that the pellet fraction contained organelles, we used cytochrome *c* oxidase activity as marker enzyme for mitochondria. Most of the cytochrome *c* oxidase activity was found in the pellet fraction (Fig. 3C, right panel). Separation of extracts derived from cells grown in medium containing 0.2% oleic acid showed a further shift of catalase activity towards the pellet fraction (Fig. 3D). Activity of CgCta1 in the various fractions was distributed corresponding to the previously observed intracellular localization of GFP–CgCta1. Together, these results showed a dual localization of *C. glabrata* catalase depending on the presence of peroxisomes.

### Phagocytosis induces GFP–CgCta1 expression

**Fig. 4:**
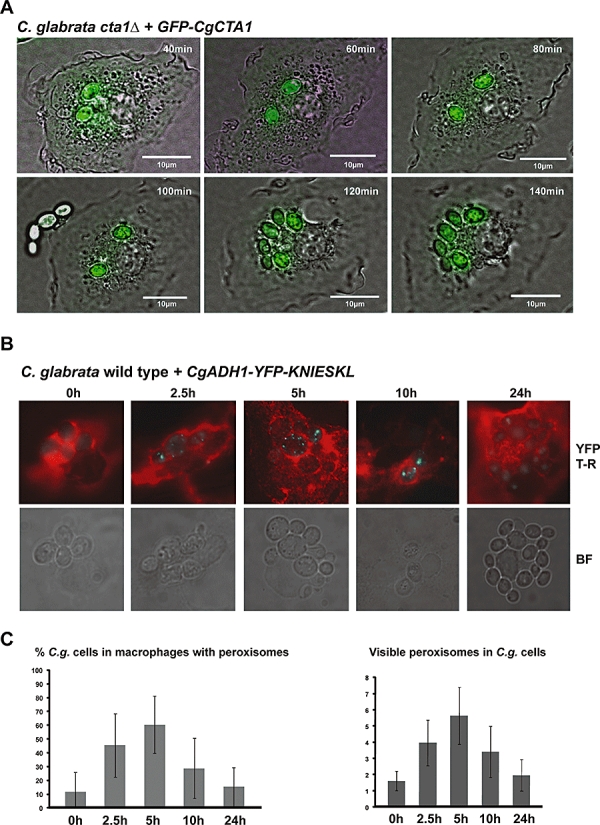
*GFP–CgCTA1* is induced upon phagocytosis and is located in both cytoplasm and peroxisomes. A. *C. glabrata* cells before and after being phagocytosed. Exponentially growing ARCg *cta1*Δ cells transformed with p*CgC–GFP–CgCTA1* were washed in PBS containing 0.1% glucose and added to macrophages in a 4:1 ratio at 37°C. Still pictures at the indicated times are shown as overlay of bright-field and fluorescence signals. B. Exponentially growing wild-type cells transformed with p*CgADH1–YFP–KNIESKL1* were washed in PBS containing 0.1% glucose and added to macrophages in a 4:1 ratio at 37°C. Cells were fixed and stained with Phalloidin Texas-Red after 0, 2.5, 5, 10 and 24 h for fluorescence microscopy. C. Percentage of phagocytosed *C. glabrata* cells with visible peroxisomes per macrophage from the total cell number of *C. glabrata* cells per macrophage after 0, 2.5, 5, 10 and 24 h (left panel). Number of visible peroxisomes within phagocytosed *C. glabrata* cells after 0, 2.5, 5, 10 and 24 h (right panel).

Fungal pathogens are exposed to a stressful environment, when they come into contact with phagocytic cells (Nicola *et al.*, 2008). The regulation and localization of GFP–CgCta1 made it useful to report the environmental conditions during phagocytosis. *C. glabrata cta1*Δ mutant cells expressing *GFP–CgCTA1* grown to exponential phase were used for infection of primary mouse macrophages. We used time-lapse live microscopy to follow the fate of individual engulfed cells (Fig. 4A). Freshly phagocytosed *C. glabrata* cells reacted to this environment with a detectable GFP–CgCta1 fluorescence signal within 40 min (Fig. 4A and Fig. S2C). Furthermore, during prolonged phagocytosis, GFP–CgCta1 accumulated in peroxisomes. To support the idea of peroxisome proliferation during phagocytosis, we followed localization of the YFP–KNIESKL fusion protein during infection of macrophages. Cells were fixed and stained for microscopy immediately after infection and after 2.5, 5, 10 and 24 h (Fig. 4B). We counted cells with visible peroxisomes per macrophage at various time points (Fig. 4C, left panel; Fig. S3). The number of cells with peroxisomes and the number of peroxisomes within these cells transiently increased, reaching a peak after 5 h (Fig. 4C). After 24 h, the vast majority of cells displayed a cytoplasmic/vacuolar YFP–KNIESKL fluorescence signal. Thus, engulfed cells show transient proliferation of peroxisomes.

### GFP–CgYap1 and CgMig1–CFP localization changes in phagocytosed cells

**Fig. 5:**
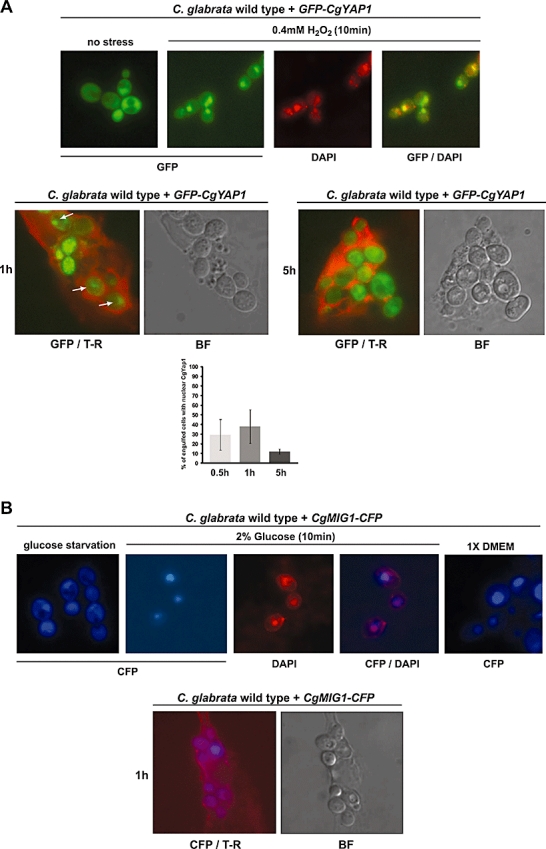
Localization of GFP–CgYap1 and CgMig1–CFP during early stage of phagocytosis. A. *C. glabrata* wild-type cells transformed with p*CgADH1–GFP–CgYAP1* were grown in synthetic medium. Cells were stressed by addition of 0.4 mM H_2_O_2_ for 10 min. Nuclei were stained with DAPI. An overlay of GFP and DAPI staining is shown in the right panel. GFP–CgYap1 visualized by fluorescence microscopy under phagocytosis conditions (lower panel). Cells were washed in PBS 0.1% glucose and added to macrophages in a 4:1 ratio and incubated at 37°C for 10 min to follow the route of tagged transcription factors. Samples were fixed and stained with Phalloidin Texas-Red. Percentage of cells with nuclear GFP–Yap1 was calculated after 30 min, 1 h and 5 h. White arrows point to nuclear GFP–CgYap1 in yeast inside the phagosome. B. *C. glabrata* wild-type cells transformed with p*CgADH1–CgMIG1–CFP* were grown in synthetic medium until glucose depletion. Cells were incubated in fresh medium containing 2% glucose for 10 min or 1× DMEM. Lower panel depicts localization of CgMig1–CFP under phagocytosis conditions. Cells were treated as described in (A).

The localization of CgCta1 suggested that engulfed *C. glabrata* cells might experience oxidative stress and/or carbon source starvation. To confirm this independently, we created additional fluorescent reporter constructs. In *S. cerevisiae*, the glucose-regulated transcriptional repressor Mig1 is rapidly exported from the nucleus in cells starved for glucose (De Vit *et al.*, 1997). *S. cerevisiae* Yap1 accumulates rapidly in the nucleus of cells exposed to mild oxidative stress (Kuge *et al.*, 1997). To preserve the localization signals of the orthologous transcription factors, CgYap1 was N-terminally fused to GFP whereas CgMig1 was C-terminally fused to CFP. To be detectable, both fusion genes were expressed from centromeric plasmids and driven by the *CgADH1* promoter. Nuclear localization was confirmed by simultaneous staining of nucleic acids with DAPI (Fig. 5A and B).

GFP–CgYap1 was located in the cytoplasm in unstressed *C. glabrata*ΔHTU cells. Upon exposure to mild oxidative stress (0.4 mM H_2_O_2_), GFP–CgYap1 rapidly accumulated in the nucleus (Fig. 5A, upper panel). The fusion gene could complement the transcription defects of the corresponding deletion mutant (our unpublished observation). Within the first hour upon engulfment, cells with nuclear GFP–CgYap1 were visible (Fig. 5A, middle panel). We determined the percentage of yeast cells with nuclear GFP–Yap1 per macrophage after 30 min, 1 h and 5 h (Fig. 5A, lower panel) and found a peak at about 1 h. The CgMig1–CFP fluorescence signal accumulated in the nucleus after addition of glucose (2%) to the medium of glucose-starved cells, and was also nuclear in the glucose-rich environment of the macrophage culture medium (DMEM) (Fig. 5B, upper panel). Immediately after phagocytosis, CgMig1–CFP accumulated in the cytoplasm and remained there constantly, indicating glucose starvation (Fig. 5B, lower panel). These data showed that within the phagosome oxidative stress is transient, whereas macrophages are highly effective in depriving the carbon source.

### Peroxisomes are transiently induced during phagocytosis

**Fig. 6:**
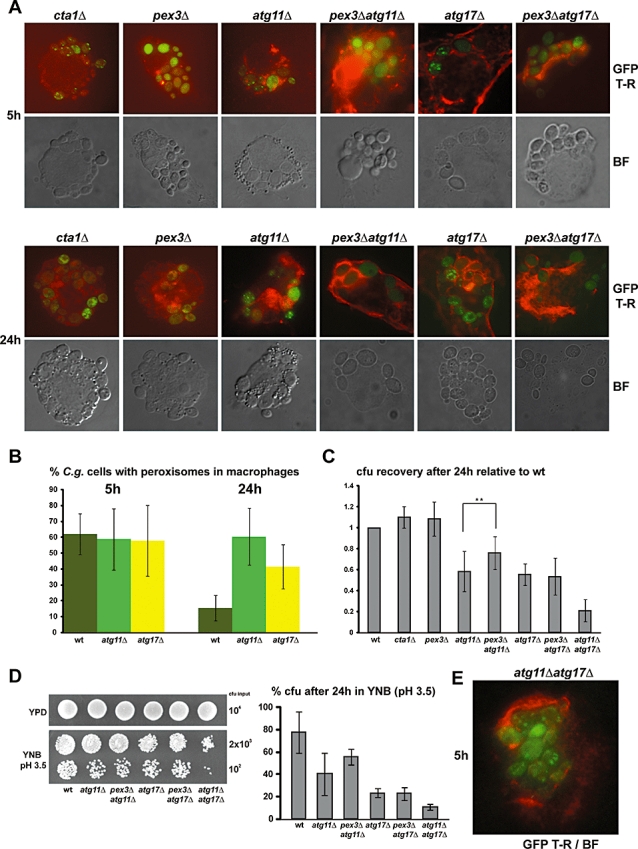
Induction and pexophagy of peroxisomes upon phagocytosis. A. Log-phase *C. glabrata* ARCg *cta1*Δ, ARCg *pex3*Δ, ARCg *atg11*Δ, ARCg *pex3*Δ*atg11*Δ, ARCg *atg17*Δ and ARCg *pex3*Δ*atg17*Δ mutant cells transformed with p*GFP–CgCTA1* were used to infect mouse macrophages in a 4:1 ratio at 37°C. Cells were fixed for microscopy after 5 and 24 h. B. Percentage of cells with visible peroxisomes after phagocytosis in macrophages after 5 and 24 h. C. Log-phase *C. glabrata* ARCg *cta1*Δ, ARCg *pex3*Δ, ARCg *atg11*Δ, ARCg *pex3*Δ*atg11*Δ, ARCg *atg17*Δ and ARCg *pex3*Δ*atg17*Δ and ARCg *atg11*Δ*atg17*Δ mutant cells were used to infect mouse macrophages in a 1:2 ratio at 37°C. The viability of the engulfed cells was assessed by hypotonic lysis of the macrophages and quantification of colony formation (cfu) on rich medium. Assays were performed in triplicate. A one-way anova was performed and *P*-values were calculated comparing the numbers of recovered colonies of the indicated strains (**P< 0.005). D. *C. glabrata* wild type, ARCg *atg11*Δ, ARCg *pex3*Δ*atg11*Δ, ARCg *atg17*Δ, ARCg *pex3*Δ*atg17*Δ and ARCg *atg11*Δ*atg17*Δ mutant cells were grown to exponential phase in rich medium; after washing with PBS supplemented with 0.1% glucose, 2 × 10^5^ cells were incubated in selective medium without nitrogen sources and glucose and pH 3.5 at 37°C. After 24 h colony formation (cfu) of mutant cells was determined. Percentage of viable cells was calculated relative to 2 h treatment. E. Log-phase ARCg *atg11*Δ*atg17*Δ mutant cells transformed with p*GFP–CgCTA1* were used to infect mouse macrophages in a 4:1 ratio at 37°C. Cells were fixed for microscopy after 5 h. Overlay of GFP/Texas-Red and BF is shown.

Peroxisome numbers declined at later stages of engulfment (Fig. 4B). In *S. cerevisiae*, key factors for pexophagy are Atg11 (Yorimitsu and Klionsky, 2005) and Atg17, which is also essential for non-selective autophagy (Cheong *et al.*, 2005). We deleted the *C. glabrata CgATG11* and *CgATG17* homologues (CAGL0H08558g, CAGL0J04686g) in wild-type (ΔHTU) and *pex3*Δ cells (Fig. S1). We investigated engulfed *C. glabrata cta1*Δ, *pex3*Δ, *atg11*Δ, *atg17*Δ, *pex3*Δ*atg17*Δ, *pex3*Δ*atg11*Δ and *atg11*Δ*atg17*Δ mutant cells expressing GFP–CgCta1 after 5 and 24 h (Fig. 6A and E). After 5 h, GFP–Cta1 was located in peroxisomes in the *cta1*Δ, *atg11*Δ and *atg17*Δ mutant cells. In contrast it accumulated in the cytoplasm of *pex3*Δ, *pex3*Δ*atg11*Δ and *pex3*Δ*atg17*Δ mutant cells. However, after 5 h, wild type, *atg11*Δ and *atg17*Δ had similar numbers of cells with peroxisomes, whereas after 24 h, peroxisomes were more abundant in *atg11*Δ and *atg17*Δ mutants (Fig. 6B). In *atg11*Δ mutants, peroxisome numbers remained constant between 5 and 24 h engulfment. *C. glabrata atg17*Δ cells displayed a slight reduction of peroxisomes after 24 h of engulfment, similar to *S. cerevisiae atg17*Δ cells during prolonged starvation conditions (Cheong *et al.*, 2005), Upon internalization, the cytoplasmic localization of CgMig1–CFP demonstrated the same glucose starvation status in the *atg11*Δ, *pex3*Δ*atg11*Δ mutants and wild type (Fig. S2B).

We investigated if the turnover of peroxisomes and mobilization of internal resources are relevant for survival during engulfment. Indeed, the *atg11*Δ and *atg17*Δ mutants had a significantly reduced viability after 24 h compared with wild type, *cta1*Δ and *pex3*Δ strains. Furthermore, in *pex3*Δ*atg11*Δ and *pex3*Δ*atg17*Δ double mutants, the absence of pexophagy might be compensated by absence of peroxisome biogenesis. Consistently, we found that the loss of Pex3 partially reversed the effect of *atg11*Δ with respect to survival during engulfment (Fig. 6C). In contrast, the double mutant *pex3*Δ*atg17*Δ did not show this phenotype, indicating a broader function for CgAtg17-dependent non-selective autophagy during engulfment. Strikingly, the *atg11*Δ*atg17*Δ double mutant, lacking both selective and non-selective autophagy, was highly sensitive to phagocytosis.

To simulate the phagosome environment *in vitro* we combined nutrient starvation and acidic pH. We incubated *C. glabrata* wild type, *atg11*Δ, *pex3*Δ*atg11*Δ, *atg17*Δ, *pex3*Δ*atg17*Δ and *atg11*Δ*atg17*Δ mutant cells in medium lacking nitrogen and carbon sources at pH 3.5 at 37°C for 24 h. The survival was determined by counting colony-forming units (cfu) after 24 h relative to 2 h treatment (Fig. 6D). In comparison with the wild type, all mutants showed diminished survival. Intriguingly, the *pex3*Δ*atg11*Δ strain survived better than the *atg11*Δ strain. Furthermore, the double mutant *atg11*Δ*atg17*Δ displayed the lowest survival rate, similar to the macrophage model. In the macrophage, after 24 h, most of the engulfed *atg11*Δ*atg17*Δ cells had lost GFP–CgCta1 fluorescence presumably due to cell death (not shown). However, after 5 h the GFP–CgCta1 fluorescence signal indicated numerous peroxisomes (Fig. 6E). These results indicated that autophagy is beneficial for survival of *C. glabrata* during engulfment in macrophages, possibly counteracting acute nutrient starvation.

## Discussion

Phagocytic cells internalize microbial cells and attack them with a range of microbicidal strategies (Chauhan *et al.*, 2006; Nicola *et al.*, 2008). Microbial pathogens have developed a number of strategies to improve their survival in the host environment (Urban *et al.*, 2006). Here we used three reporters (CgCta1, CgYap1 and CgMig1) to visualize aspects of the response of the human fungal pathogen *C. glabrata* to macrophage engulfment. We found that *C. glabrata* cells engulfed by primary mouse macrophages suffer from transient oxidative stress, show signs of carbon source starvation, and transiently induce peroxisomes. Our results revealed that the recycling of internal resources, especially peroxisomes, plays an important protective role for *C. glabrata* during engulfment in the phagosome.

The presence and/or proliferation of peroxisomes in fungal cells points to adjustment of carbon metabolism. We demonstrated accumulation of peroxisomes in *C. glabrata* during growth on non-fermentable carbon sources and during engulfment in macrophages. Peroxisomes were visualized using two fluorescent reporter constructs GFP–CgCta1 and YFP–KNIESKL and further confirmed by other criteria. They were induced on medium containing ethanol and oleic acid as carbon source. Furthermore, peroxisomes were dependent on the *CgPEX3* gene, a peroxisomal integral membrane protein, whose orthologue in *S. cerevisiae* is essential for peroxisomal biogenesis (Hohfeld *et al.*, 1991). Peroxisomal catalases such as *S. cerevisiae* Cta1 (Simon *et al.*, 1991) are scavengers of hydrogen peroxide generated during peroxisomal β-oxidation. We find that *C. glabrata* catalase expression is regulated by oxidative stress and carbon source, and its intracellular localization correlates with the presence of peroxisomes. This combines the regulation of both yeast catalases. The *CgCTA1* gene lacks synteny with the yeast *CTA1* gene and other fungal catalases (Gordon *et al.*, 2009). It is tempting to speculate that the shuffling of the *C. glabrata* genome fostered the accumulation of regulatory elements for oxidative stress and carbon source response.

In a phagocytosis model using bone marrow-derived mouse macrophages, GFP–CgCta1 expressed under the control of the *CgCTA1* promoter was induced in the earliest stages after internalization. This could be due to oxidative stress or acute carbon starvation. Intracellular localization of two other fluorescent reporters (CgYap1 and CgMig1) supported rather low oxidative stress load and starvation for glucose of engulfed *C. glabrata* cells. High-level expression was necessary for detection of GFP–transcription factor fusions and could potentially interfere with signalling. However, both factors are tightly regulated by post-translational modifications and thus buffered for expression level. We found that in a population of engulfed cells a minor fraction displayed signs of acute oxidative stress. This is consistent with other reports. Only a small portion of *C. albicans* cells derived from mouse kidneys displayed an acute oxidative stress response when examined for *CaCTA1* expression (Enjalbert *et al.*, 2007).

The *C. glabrata* transcriptional response might have been selected to the specific conditions of phagocytosis. Microarray data indicated induction of a group of about 30 genes by both oxidative stress and glucose starvation (Roetzer *et al.*, 2008). Moreover, phagocytosed *C. glabrata* cells induce genes involved in gluconeogenesis, β-oxidation, glyoxylate cycle, and transporters for amino acids and acetate (Kaur *et al.*, 2007). Induction of peroxisomes after internalization by macrophages indicated adjustment of metabolism within the phagosome. Cells utilizing non-fermentable carbon sources, e.g. fatty acids or ethanol, require peroxisomal β-oxidation and the partly peroxisomal glyoxylate cycle. The induction of non-fermentative carbon metabolism genes is beneficial for the survival of *C. albicans* (Lorenz *et al.*, 2004; Barelle *et al.*, 2006). In a mouse infection model, Fox2, the second enzyme of the β-oxidation pathway, and isocitrate lyase (Icl1) an enzyme of the glyoxylate cycle, were required for *C. albicans* virulence (Lorenz and Fink, 2001; Piekarska *et al.*, 2006). However, *C. albicans* mutants defective in the import receptor of PTS1-targeted peroxisomal proteins, CaPex5, displayed no attenuation of virulence (Piekarska *et al.*, 2006). The survival of *C. glabrata* devoid of peroxisomes in a *pex3*Δ mutant was not compromised in our infection model. Also, *C. neoformans pex1*Δ deletion mutants were not attenuated for virulence (Idnurm *et al.*, 2007). These data support the view that peroxisomes are not a major virulence determinant. Instead, the peroxisomal metabolic pathways, which can function to sufficient extent in the cytosol, appear to contribute to virulence.

In engulfed *C. glabrata* cells peroxisome numbers declined at later time points. Also at later time points GFP–CgCta1 accumulated partly in the cytosol. Peroxisomes are not known to export proteins, thus the cytosolic fluorescence was most probably due to *de novo* synthesis or peroxisome turnover. Peroxisomes are degraded by pexophagy, a selective autophagic pathway (Hutchins *et al.*, 1999; Farre and Subramani, 2004). In *S. cerevisiae*, mutants lacking Atg11 and Atg17 had a severe delay of pexophagy (Kim *et al.*, 2001; Cheong *et al.*, 2005; 2008). In *C. glabrata*, we found that mutants lacking *atg11*Δ or *atg17*Δ had reduced survival in macrophages and *in vitro* during starvation. Moreover, the *C. glabrata* double mutant *atg11*Δ*atg17*Δ displayed a striking additive decrease of survival. In *S. cerevisiae*, the *atg11*Δ*atg17*Δ double mutant strain did not contain any detectable autophagic bodies and had a severe autophagy defect (Cheong *et al.*, 2008). We suggest that *C. glabrata atg11*Δ*atg17*Δ is unable to induce autophagic processes in order to sustain prolonged phagocytosis. Notably, homologues of proteins of the autophagy core machinery have been found from yeast to mammals, but both Atg11 and Atg17 are not conserved and might be a target for antifungal drugs (reviewed by Xie and Klionsky, 2007).

Autophagy is required for *C. neoformans* virulence (Hu *et al.*, 2008). Furthermore, *C. neoformans* genes involved in autophagy, peroxisome function and lipid metabolism became also induced during infection (Fan *et al.*, 2005). *C. neoformans* could escape from macrophages through extrusions of the phagosome, without killing the phagocytic cell (Alvarez and Casadevall, 2006). It has been suggested that this is a pathway for dissemination within the host. Therefore, survival in the macrophage indirectly contributes to virulence. A *C. albicans* mutant lacking Ca*ATG9* was defective for autophagy, but nevertheless was able to kill macrophages (Palmer *et al.*, 2007). In contrast to *C. albicans*, *C. glabrata* is trapped inside the phagosome. In *C. glabrata pex3*Δ*atg11*Δ cells, we found the sensitivity of *atg11*Δ partially reversed. We suggest from this genetic observation that autophagy of peroxisomes is beneficial for engulfed *C. glabrata* cells. *C. glabrata pex3*Δ*atg17*Δ mutants did not display this effect. Selective pexophagy, which is affected in both *atg11*Δ and *atg17*Δ mutants, might help to mobilize intracellular resources during prolonged engulfment. *S. cerevisiae* uses autophagy to recycle proteins to overcome nitrogen starvation (Onodera and Ohsumi, 2005). Autophagic processes, such as pexophagy, are contributing to virulence of important fungal plant pathogens (Veneault-Fourrey *et al.*, 2006; Asakura *et al.*, 2009). However, an *A. fumigatus* mutant strain lacking Atg1 also remained virulent (Richie *et al.*, 2007). Thus the role of autophagy for fungal pathogens is also dependent on their morphology (Palmer *et al.*, 2008).

Beside the carbon and nitrogen starvation conditions inside the phagosome, other restrictions, such as pH, hydrolytic enzymes and antimicrobial peptides, might act in a synergistic manner. We infer from the phenotype of our autophagy mutants that macrophage engulfment is essentially a starvation situation in combination with acidic pH. Acidification of the phagosome aids the destruction of some microbes, but it might also contribute to the escape of others. For example, lysosomal acidification induced germ tube formation of *C. albicans* and therefore contributed to its escape from the macrophage (Kaposzta *et al.*, 1999). The observed oxidative stress response of *C. glabrata* might result from a switch of metabolism rather than a macrophage-derived oxidative burst. It has been reported that in *S. cerevisiae*, a shift to oleic acid as carbon source induced a specific Yap1-dependent subset of oxidative stress response genes (Koerkamp *et al.*, 2002). However, we believe that the importance of autophagy for survival suggests a starvation situation. Furthermore, in our model system, the transient induction and degradation of peroxisomes is not supporting substantial metabolism in the phagosome.

Our results demonstrate that monitoring of the intracellular localization of proteins tagged with fluorescent reporters is a highly informative tool to reveal intracellular signalling and metabolic conditions. Here we show that the macrophage is efficiently depriving engulfed *C. glabrata* cells from nutrient sources. Autophagic processes, prolonging the survival of engulfed cells, are potentially aiding the dissemination of *C. glabrata* and the establishment of infection.

## Experimental procedures

### Yeast strains and plasmids

Yeast strains used in this study are listed in Table 1. Rich medium (YPD), synthetic medium (SC) and yeast nitrogen base medium (YNB) without amino acids and ammonium sulfate were prepared as described elsewhere (Current Protocols in Molecular Biology; Wiley). All strains were grown at 30°C or 37°C as indicated*.* Oleate medium contained 0.2% oleic acid, 0.3% yeast extract, 0.5% peptone and 0.5% KH_2_PO_4_ (pH 6). Oleate plates were incubated at 37°C for 7 days. Glucose concentration between 0.5% and 0.03% (w/v) was determined using the Freestyle mini (Abbott). To assess viability of cells during starvation (Fig. 6D), cfu were determined by spreading on rich medium, usually after 2 h of incubation at 37°C and after the indicated time (24 h). Oligonucleotides used in this study are listed in Table S1. *C. glabrata* strains AR*Cg cta1*Δ, AR*Cg pex3*Δ, AR*Cg atg11*Δ, AR*Cg pex3*Δ*atg11*Δ, AR*Cg atg17*Δ, AR*Cg pex3*Δ*atg17*Δ and ARCg *atg11*Δ*atg17*Δ were obtained by replacing the ORFs with the *S. cerevisiae URA3* gene or *HIS3* gene generated by genomic integration. Knockout cassettes were synthesized using fusion PCR according to Noble (Noble and Johnson, 2005) from the plasmids pRS316 and pRS313 (Sikorski and Hieter, 1989) with the oligonucleotides CTA1-1 to 6, PEX3-1 to 6, ATG11-1 to 6 and ATG17-1 to 6. Correct genomic integration was verified by genomic PCR (primer series Ctrl) followed by Southern analysis using probes generated with primers CTA1-4/CTA1-6, PEX3-1/PEX3-3, ATG11-4/ATG11-6 and ATG17-1/ATG17-3 or ATG17-4/ATG17-6. Probes for Southern and also for Northern analysis (CTA1-5/CTA1-3 and ACT1-5/ACT1-3) were amplified by PCR from genomic DNA.

**Table 1:** Strains used in this study.

*C. glabrata* strain	Genotype	Source
ΔHTU	*his3*Δ*trp1*Δ*ura3*Δ	Kitada *et al.* (1996)
ΔHT6	*his3*Δ*trp1*Δ	Kitada *et al.* (1996)
ARCg *cta1*Δ	*his3*Δ*trp1*Δ*ura3*Δ*cta1*Δ::*ScURA3*	This study
ARCg *pex3*Δ	*his3*Δ*trp1*Δ*ura3*Δ*pex3*Δ::*ScURA3*	This study
ARCg *atg11*Δ	*his3*Δ*trp1*Δ*ura3*Δ*atg11*Δ::*ScURA3*	This study
ARCg *pex3*Δ*atg11*Δ	*his3*Δ*trp1*Δ*ura3*Δ*pex3*Δ::*ScURA3 atg11*Δ*::ScHIS3*	This study
ARCg *atg17*Δ	*his3*Δ*trp1*Δ*ura3*Δ*atg17*Δ::*ScURA3*	This study
ARCg *pex3*Δ*atg17*Δ	*his3*Δ*trp1*Δ*ura3*Δ*pex3*Δ::*ScURA3 atg17*Δ*::ScHIS3*	This study
ARCg *atg11*Δ*atg17*Δ	*his3*Δ*trp1*Δ*ura3*Δ*atg11*Δ::*ScURA3 atg17*Δ*::ScHIS3*	This study

Plasmids used in this study are listed in Table 2. To generate pGEM–ACT–CgCTA1, 1800 base pairs of the CgCTA1 promoter were inserted as a SphI/NotI PCR product obtained with primers CTAPro-up and CTAPro-down into the plasmid pGEM–ACT (Gregori *et al.*, 2007). The coding sequence for CgCTA1 was amplified from genomic DNA using primers CTA-up-Not and CTA-down-Nsi, cut and inserted as a NotI/NsiI fragment. GFP was inserted as a NotI/NotI fragment at the N-terminus of CgCTA1. To generate pYFP–KNIESKL YFP was inserted as a NotI/NotI fragment obtained by PCR with primers YFP–Not-Start and YFP–SKL-Stop into the plasmid pGEM–ACT–CgADH1 (Roetzer *et al.*, 2008). CgYAP1 was amplified using primers CgYap5/CgYap3 containing a NotI or a NsiI site; GFP was inserted as NotI/NotI fragment into the plasmid pGEM–ACT–CgADH1 at the N-terminus of CgYAP1. CgMIG1 was amplified using primers Mig1-5sac/Mig1-3nco and inserted into NcoI and SacII cut pGEM–ACT–CgADH1–MSN2–CFP (Roetzer *et al.*, 2008). All cloned PCR fragments used in this study were controlled by sequencing.

**Table 2:** Plasmids used in this study.

Plamid	Genotype	Source
p*RS316*	CEN6, ARSH4, *ScURA3*	Sikorski and Hieter (1989)
p*RS313*	CEN6, ARSH4, *ScHIS3*	Sikorski and Hieter (1989)
p*ACT14*	ARS, CEN and *TRP1* marker from *C. glabrata*	Kitada *et al.* (1996)
p*GEM–ACT*	ARS, CEN and *TRP1* marker from *C. glabrata*	Gregori *et al.* (2007)
p*CgC–GFP–CgCTA1*	CgCTA1–GFP*–CgCTA1* (*CgCTA1p*: SphI/NotI, *CgCTA1* ORF NotII/NsiI, GFP NotI/NotI) *CgTRP1*	This study
p*CgC–CgCTA1*	CgCTA1*–CgCTA1* (SphI/NotI and NotII/NsiI); *CgTRP1* marker	This study
p*CgCADH1–YFP–KNIESKL*	CgCADH1*–YFP–KNIESKL* (NotI/NotI fragment); *CgTRP1*	This study
p*CgADH1–CgMSN2–CFP*	*CgADH1–CgMSN2–CFP* (*CgADH1p*: SphI/SacII and *CgMSN2*: SacII/NsiI); *CgTRP1*	Roetzer *et al.* (2008)
p*CgADH1–CgMIG1–CFP*	*CgADH1–CgMIG1–CFP* (*CgMIG1*: SacII/NcoI); *CgTRP1*	This study
p*CgADH1–GFP–CgYAP1*	*CgADH1–GFP–CgYAP1* (*CgYAP1*: NotII/NsiI); *CgTRP1*	This study

### Catalase and cytochrome *c* oxidase assay

Crude extracts were prepared by breakage of yeast cells with glass beads. Catalase activity was assayed spectrophotometrically at 240 nm as described in Durchschlag *et al.* (2004); protein concentrations were assayed at 280 nm. For the cytochrome *c* oxidase assay, 0.5 g l^−1^ Sodium dithionite was added to reduce cytochrome *c* (0.1 mg ml^−1^) solution. Cytochrome *c* has a sharp absorption band at 550 nm in the reduced state. Absorption spectra of cytochrome *c* were recorded between 410 and 570 nm. Five minutes after addition of crude extracts, spectra were measured to determine the oxidized state of cytochrome *c* (Lemberg, 1969).

### Separation of organelles

Cells were re-suspended in washing buffer (20 mM Hepes pH 7.4, 50 mM NaCl, 0.6 M sorbitol), incubated with protease inhibitor PMSF and broken using glass beads. The supernatant was centrifuged for 12 min at 6900 rcf to separate (post-mitochondrial) supernatant and the organellar pellet.

### Northern and Southern blot analysis

RNA extraction and separation followed essentially the described protocol (Current Protocols in Molecular Biology; Wiley). Hybridization of [α-^32^P]-dATP-labelled probes occurred overnight in hybridization buffer (0.5 M Sodium phosphate buffer pH 7.2/7% SDS/1 mM EDTA) at 65°C. For DNA extraction, 10 ml yeast cells (grown to an OD_600_ = 6) were collected, washed once and re-suspended in Lysis buffer (2% Triton X-100/1% SDS/100 mM NaCl/10 mM Tris pH 8/1 mM EDTA). Genomic DNA was isolated by PCI (phenol/chloroform/isoamyl alcohol) extraction. Digestion of 10 μg of genomic DNA was performed overnight with XcmI for *CgPEX3*, EcoRV for *CgCTA1* and ClaI/NcoI for *CgATG11* (5 U μg^−1^ DNA). The labelled probes were hybridized overnight in hybridization buffer at 65°C*.* Signals were visualized by autoradiography.

### Microscopy

GFP-fluorescence microscopy was performed as described previously (Görner *et al.*, 1998). GFP was visualized in live cells without fixation. All cells were monitored using a Zeiss Axioplan 2 fluorescence microscope. Images were captured with a Spot Pursuit (Sony) CCD camera using Spotbasic software. Time-lapse microscopy was performed on an Olympus cell-imager system (IX81 inverted microscope) equipped for cell culture observation. Cells were incubated in a glass chamber at 37°C connected to an active gas mixer (Ibidi, Martinsried, Germany). Pictures were taken with a Hamamatsu ORCA-ER camera and analysed using cell^M^&cell^R^ software (Olympus). Nomarski contrasted, bright-field microscopy pictures are indicated as BF. Quantification and statistical analysis of peroxisomes in *C. glabrata* cells (Figs 2D, 4C and 6B) have been added in Fig. S3.

### Macrophage cell culture

Primary bone marrow-derived macrophages (BMDMs) were obtained from the femur bone marrow of 6- to 10-week-old C57Bl/6 mice. Cells were cultivated in DMEM supplemented with 10% FCS in the presence of L cell-derived CSF-1 as described (Baccarini *et al.*, 1985). Mice were housed under specific pathogen-free conditions. For infection assays, BMDMs were seeded at 5 × 10^5^ cells per dish in 3.5 cm dishes containing medium without antibiotics. Log-phase *C. glabrata* cells were washed with PBS supplemented with 0.1% glucose and added to macrophages in a 4:1 ratio and incubated at 37°C. For microscopy, cells were fixed with 2% formaldehyde for 5 min. After washing with PBS, cells were incubated in 1% Triton X-100 for 1 min. After washing with PBS, cells were dyed with Phalloidin Texas-Red for 30 min. Coverslips were fixed to slides with Mowiol. For cfu assays, BMDMs were seeded at 2 × 10^5^ cells per dish. Exponentially growing *C. glabrata* cells were washed with PBS supplemented with 0.1% glucose and added to macrophages in a 1:2 ratio and incubated at 37°C. After 45 min, cells were washed three times with PBS to remove not phagocytosed yeast cells and fresh medium was added. At the indicated times, deionized water was added to lyse macrophage cells. *C. glabrata* cells were spread on YPD plates, colonies were counted after incubation at 37°C for 2 days.

## Supporting information

**Fig. S1.** Southern blot analysis of *cta1*Δ, *pex3*Δ, *atg11*Δ and *pex3*Δ *atg11*Δ deletion strains. Both *CgCTA1* and *CgPEX3* were replaced by *ScURA3. CgATG11* was replaced by *ScURA3* in the single mutant and by *ScHIS3* in the double mutant ARCg *pex3*Δ *atg11*Δ. *CgATG17* was replaced by *ScURA3* in the single mutant and by *ScHIS3* in the double mutants ARCg *pex3*Δ *atg17*Δ and ARCg *atg11*Δ *atg17*Δ. Amplified probes and chromosomal restriction enzyme locations are indicated. Chromosomal DNA derived from ARCg *cta1*Δ digested with EcoRV and ARCg *pex3*Δ with XcmI resulted in shortened fragments relative to wild type. Digestion of chromosomal DNA from the ARCg *atg11*Δ strain with NcoI and from the ARCg *pex3*Δ *atg11*Δ double mutant strain with ClaI led to shorter fragments in both cases, since *CgATG11* contains neither a NcoI site nor a ClaI site. Picture is a composite of two exposures of the same blot, due to different amounts of DNA (lower panel). Chromosomal DNA derived from ARCg *atg17*Δ digested with AflIII and ARCg *pex3*Δ *patg17*Δ or ARCg *atg11*Δ *atg17*Δ with NdeI resulted in shortened fragments.

**Fig. S2.** Glucose depletion leads to induction of *GFP–CgCTA1* and cytoplasmic localization of CgMig1–CFP.

A. *GFP–CgCTA1* is induced upon glucose depletion and is located in the cytoplasm. *C. glabrata* ARCg *cta1*Δ cells transformed with p*CgC–GFP–CgCTA1* were grown in rich medium with glucose to exponential phase and washed twice and incubated in rich medium including 0.5% glucose. Every 10 min concentration of glucose was determined (see *Experimental procedures*) and samples were fixed for microscopy. GFP fluorescence was visible at about 40 min after glucose exhaustion.

B. Localization of CgMig1–CFP in ARCg *atg11*Δ and ARCg *pex3*Δ *atg11*Δ mutants during internalization by macrophages. CgMig1–CFP was visualized by fluorescence microscopy under phagocytosis conditions. *C. glabrata* wild-type cells transformed with p*CgADH1–CgMIG1–CFP* were grown to exponential phase, washed in PBS 0.1% glucose and added to macrophages in a 4:1 ratio and incubated at 37°C for 1 h. Samples were fixed and stained with Phalloidin Texas-Red.

C. *GFP–CgCTA1* is induced upon phagocytosis. Still pictures from time-lapse analysis are shown as overlay of bright-field and fluorescence signals. Exponentially growing ARCg *cta1*Δ cells transformed with p*CgC–GFP–CgCTA1* were washed in PBS containing 0.1% glucose and added to macrophages in a 4:1 ratio at 37°C.

**Fig. S3.** Quantification details.

**Table S1.** Oligonucleotides used.
