# Supplementary figures and images for: Autophagy supports Candida glabrata survival during phagocytosis
Source: Cell Microbiol. 2009 Oct 26;12(2):199–216. doi: 10.1111/j.1462-5822.2009.01391.x (PMC2816358; doi:10.1111/j.1462-5822.2009.01391.x)

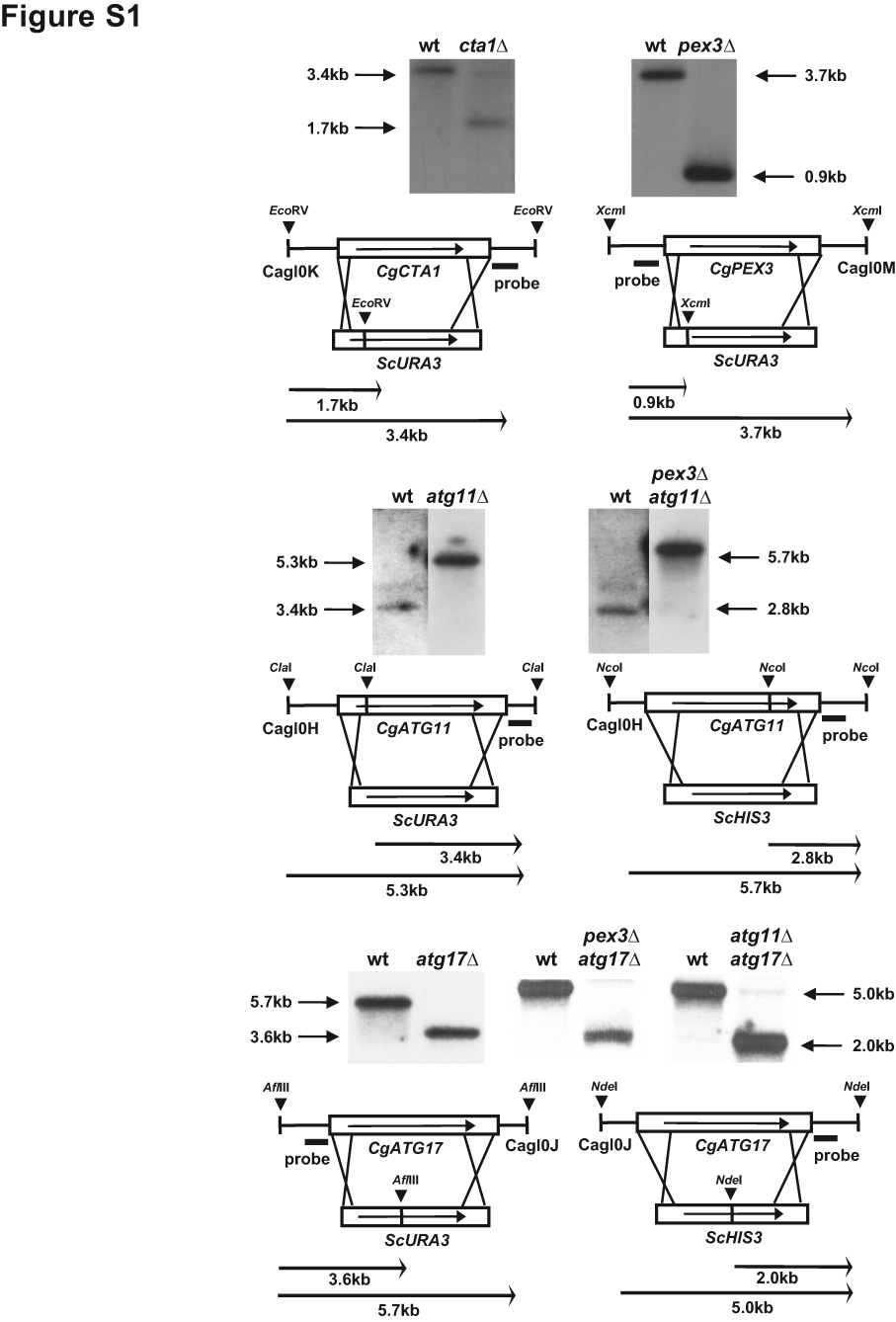

Supplement: Supplementary file 1 [file cmi0012-0199-SD1.tif]

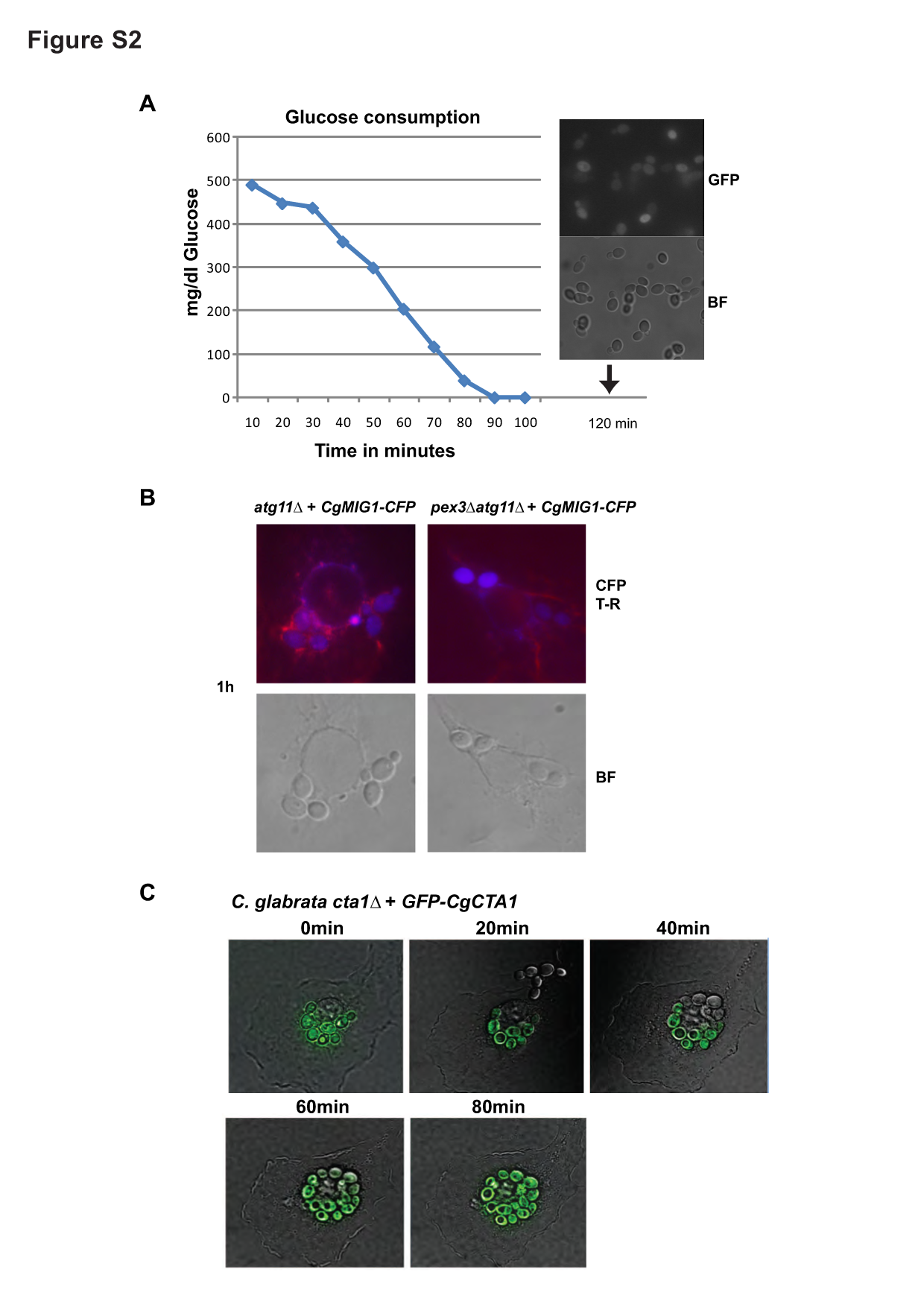

Supplement: Supplementary file 2 [file cmi0012-0199-SD2.tif]

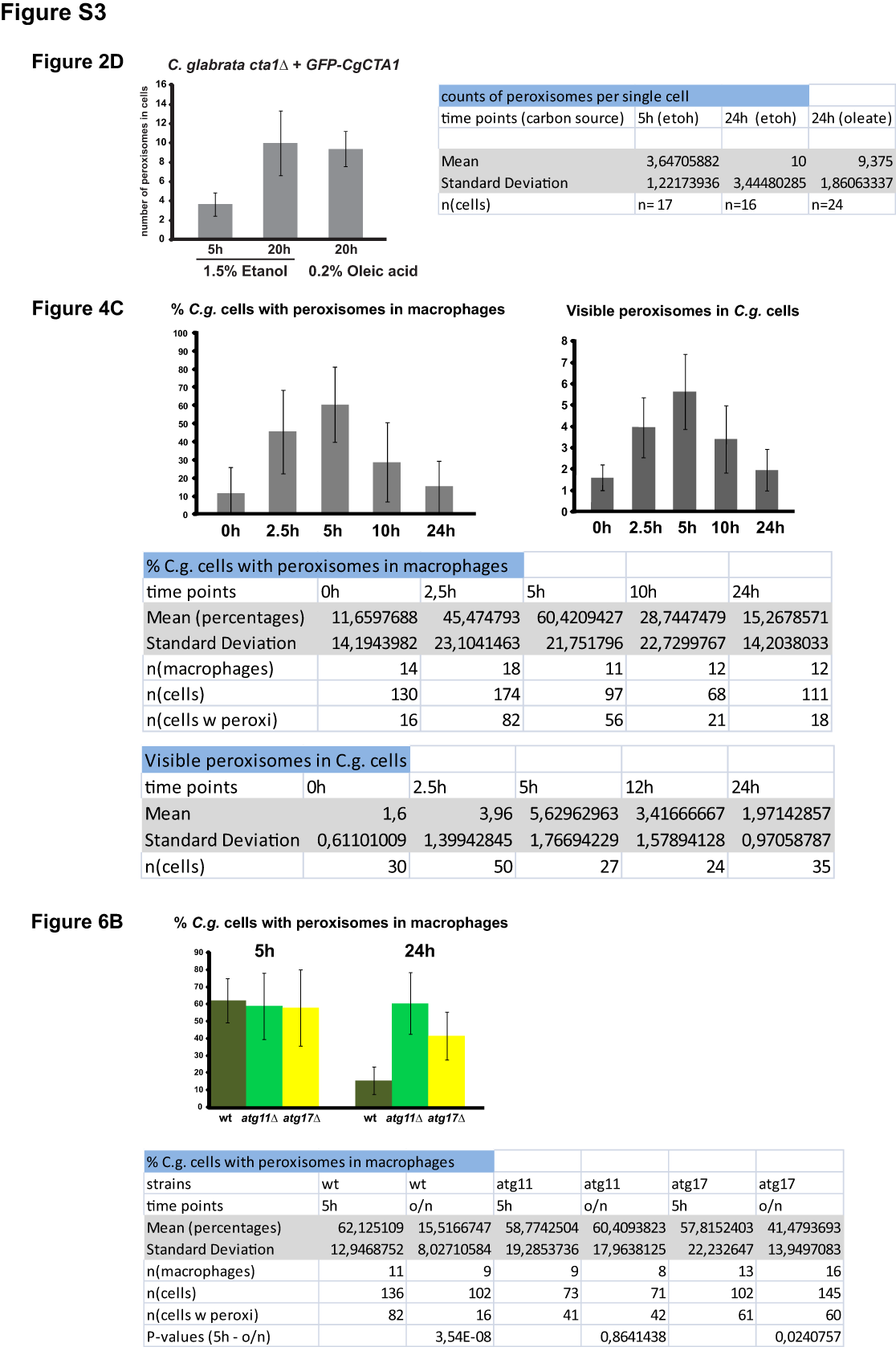

Supplement: Supplementary file 3 [file cmi0012-0199-SD3.tif]
