# Supplementary material for: Autophagy supports Candida glabrata survival during phagocytosis
Source: Cell Microbiol. 2009 Oct 26;12(2):199–216. doi: 10.1111/j.1462-5822.2009.01391.x (PMC2816358; doi:10.1111/j.1462-5822.2009.01391.x)
Supplement: Supplementary file 4 [file cmi0012-0199-SD4.doc]

**Supplemental Table S1**

| **Name** | **Sequence** |
| --- | --- |
| CTAPro-up | GGATTGCATGCTCAATATTGCCTTGTTGGCA |
| CTAPro-down | ATGTCGACTGCGGCCCATGGTTTTTTTCAATTGTGGGAAGTTATC |
| CTA-up-Not | AGATGGGCGGCCGCAATCCAACTAACACTTCCGATGTTAGAGG |
| CTA-down-Nsi | AAGTCGACAACATCAATGCATCCAGTGAC |
| YFP-Not-Start | GCCTGTGCGGCCGCATGGTGAGC |
| YFP-SKL-Not-Stop | GTCACAGCGGCCGCATTACAGTTTACTTTCAATGTTTTTGCGCCCGCTCTTGTACAGCT |
| CTAPro-Seq | CAACATTATAACGCT |
| GFP3´-Seq | TGCTGGAGTTCGTGA |
| ACT1-5 | ATGTGTAAGGCCGGTTTC |
| ACT1-3 | AGGAAGATTGAGCAGCGG |
| CTA1-5 | ATGTCCGCTAATCCAACT |
| CTA1-3 | GCTTCATCATTGGTCAAG |
| CTA1-1 | TACCTTGGAACTTGGGATAA |
| CTA1-2 | CCGCTGCTAGGCGCGCCGTGAGATTGTACTGAGAGTGCAC |
| CTA1-3 | CACGGCGCGCCTAGCAGCGGCAATTGTGGGAAGTTATCTA |
| CTA1-4 | GTCAGCGGCCGCATCCCTGCGTGCGCTTTTGAACCACGTA |
| CTA1-5 | GCAGGGATGCGGCCGCTGACCTGTGCGGTATTTCACACCG |
| CTA1-6 | GCGGTACAATGGACAACATC |
| 5´CTA1-Ctrl1 | TGCATGAAGGAGAGA |
| URA-Ctrl2 | TGCTGGCCGCATCTTCT |
| 3´CTA1-Ctrl3 | CCTGCGTTGTTAACT |
| URA-Ctrl4 | TAGTCCTGTTGCTGCCA |
| PEX3-1 | TGCGTCTTCAACAGCGGTAA |
| PEX3-3 | CACGGCGCGCCTAGCAGCGGCGCTGTGCCTATCACTAGA |
| PEX3-4 | GTCAGCGGCCGCATCCCTGCAGAATGGGGATACATAGTG |
| PEX3-6 | AGGGTTGAGAGACGTTGTCT |
| PEX3-Ctrl5 | TGTCCAGGAACTGCCTGGCA |
| PEX3-Ctrl3 | GTTGCGATAAACTCGTGAAG |
| CgYap5 | ACTAGGCGGCCGCCCAGGAATGGCTGAGGTGGATAACGG |
| CgYap3 | GACGATGCATTTATTAAGACATGTGCTTAT |
| MIG1-5sac | GCTACCATGGTGCCGCCACCAGTTGGAAATTGCAAGT |
| MIG1-3nco | CGAGTCCGCGGATGTCTGCATCAGCGAGTCC |
| ATG11-1 | CCGTGTCCGCGGTATGCGCT |
| ATG11-3 | CACGGCGCGCCTAGCAGCGGTGTGTCAGCGCGATGGTATG |
| ATG11-4 | GTCAGCGGCCGCATCCCTGCAATGAACTCTCGATACAGAA |
| ATG11-6 | AATGATTTAATTAAAGAGA |
| ATG11-Ctrl5 | GCTCACACAACAACACATC |
| ATG11-Ctrl3 | GCCGATGAACAATCTATGGC |
| ATG17-1 | GCGTAACAAGCACATATACA |
| ATG17-3 | CACGGCGCGCCTAGCAGCGGTGTATATGTGCTTGTTACGC |
| ATG17-4 | GTCAGCGGCCGCATCCCTGCACACTGAGGCAGATCACTAG |
| ATG17-6 | GAAACCGTATTGATATCAGC |
| ATG17-Ctrl5 | CAAAGTATTCAATTGGGT |
| ATG17-Ctrl3 | GTGAGCTATCTGGAACAG |
